# Development and validation of a prognostic nomogram for gastrointestinal stromal tumors in the postimatinib era: A study based on the SEER database and a Chinese cohort

**DOI:** 10.1002/cam4.6240

**Published:** 2023-06-17

**Authors:** Shu Wang, Yuhao Wang, Jialin Luo, Haoyuan Wang, Yan Zhao, Yongzhan Nie, Jianjun Yang

**Affiliations:** ^1^ Department of Digestive Surgery Xi Jing Hospital, The Fourth Military Medical University Xi'an China; ^2^ State Key Laboratory of Cancer Biology, National Clinical Research Center for Digestive Diseases and Xijing Hospital of Digestive Diseases The Fourth Military Medical University Xi'an China

**Keywords:** gastrointestinal stromal tumor, nomogram, prognostic factors, SEER analyses

## Abstract

**Background:**

After the standardization, recording and follow‐up of imatinib use that significantly prolongs survival of gastrointestinal stromal tumors (GISTs), a comprehensive reassessment of the prognosis of GISTs is necessary and more conductive to treatment options.

**Methods:**

A total of 2185 GISTs between 2013 and 2016 were obtained from the Surveillance, Epidemiology, and End Results database and comprised our training (*n* = 1456) and internal validation cohorts (*n* = 729). The risk factors extracted from univariate and multivariate analyses were used to establish a predictive nomogram. The model was evaluated and tested in the validation cohort internally and in 159 patients with GIST diagnosed between January 2015 and June 2017 in Xijing Hospital externally.

**Results:**

The median OS was 49 months (range, 0–83 months) in the training cohort and 51 months (0–83 months) in the validation cohort. The concordance index (C‐index) of the nomogram was 0.777 (95% CI, 0.752–0.802) and 0.7787 (0.7785, bootstrap corrected) in training and internal validation cohorts, respectively, and 0.7613 (0.7579, bootstrap corrected) in the external validation cohort. Receiver operating characteristic curves and calibration curves for 1‐, 3‐, and 5‐year overall survival (OS) showed a high degree of discrimination and calibration. The area under the curve showed that the new model performed better than the TNM staging system. In addition, the model could be dynamically visualized on a webpage.

**Conclusion:**

We developed a comprehensive survival prediction model for assessing the 1‐, 3‐ and 5‐year OS of patients with GIST in the postimatinib era. This predictive model outperforms the traditional TNM staging system and sheds light on the improvement of the prognostic prediction and the selection of treatment strategies for GISTs.

## 1 INTRODUCTION

Gastrointestinal stromal tumor (GIST) is frequently considered a histological subtype of sarcoma and accounts for about 18% of all histological subtypes.^1^ It can originate along the entire digestive tract and has an annual incidence of 6–22/10^6^ people in the population, with variations by region and over time.^2^, ^3^, ^4^ GIST is driven by genetic mutations with distinct signatures, with KIT mutations presenting in 60%–70% of cases and platelet‐derived growth factor receptor alpha (PDGFRA) in 10%–15%.^5^, ^6^, ^7^ Typically, the clinical presentation is gastric or intestinal masses. Bleeding, anemia, abdominal pain, and/or ileus are ordinary symptoms. Treatment management of GIST includes surgery and systematic systemic therapy, which requires the multidisciplinary collaboration of oncologists, surgeons, radiation therapists, and nuclear medicine and molecular pathology teams. Surgery is generally the first clinical option for localized GIST.^8^ The effect of surgical resection on localized GIST prognosis has been widely recognized. However, in patients with advanced GISTs, one of these cases is with metastatic diseases, cannot initially undergo surgery, and require initial treatment with medications. One is that surgical intervention is not initially possible for giant localized tumors and/or those difficult to remove because of potential complications due to adjacent organs.^9^ In the past 20 years, the survival of GIST patients has been remarkably prolonged by the introduction of tyrosine kinase inhibitors (TKIs) targeting gene mutations, mostly KIT or PDGFRA. TKIs increased the median overall survival (OS) of patients with advanced tumors from 18 months to >5 years.^8^, ^10^ Compared with 1‐year adjuvant medication, 3‐year adjuvant medication with imatinib averted ~50% of deaths during a 10‐year postoperative follow‐up period.^11^ In the advanced cases, wherein, tumors are unresectable and metastatic, TKIs can increase the R0 resection rate, manage the micro‐metastasis, and safely decrease postoperative recurrence and metastasis, thereby extending OS and disease‐free survival.^12^ Although neoadjuvant therapy can improve R0 resection rates, there are some risks associated with continuous and simple pharmacotherapy. In the absence of close monitoring, tumors may insidiously progress owing to secondary resistance to the initial medication and may not be surgically resectable by the time this concern is identified.^13^ Therefore, physicians need to preemptively identify patients who have a poor prognosis and are likely to obtain limited benefit from simply subsequent medications. This may lead to identifying a tumor while it is still surgically resectable and offers the hope of potentially better outcomes.

Existing methods of assessing patients' conditions are mostly restricted to limited variable inclusion. The TNM staging system, which is widely applied for prognostic evaluation, only considers tumor size, depth of invasion, lymph node involvement, and distant metastasis. As lymph node involvement is rare in GIST,^10^ the practical value of TNM staging is weakened further. The modified NIH criteria published in 2008 recommended that the mitotic index and tumor dimensions should be taken into consideration.^14^ Then, the Armed Forces Institute of Pathology criteria further recommended that the primary tumor location should be taken into consideration.^15^ However, predicting the biological behavior of a GIST based on pathological features alone is well known to be difficult, and clinical dispositions, such as cancer‐directed surgical resection and chemotherapy, significantly affect the outcomes. Published GIST prognostic models have had limited utility with respect to the restrictions mentioned and have failed to address the role of dispositions and resolve patient stratification.^16^, ^17^, ^18^

The SEER database subsidized by the US National Cancer Institute is publicly available and contains superfluous data for practically all cancers.^19^ The most successful example of TKI, imatinib, reportedly performed well in a randomized, open‐label, multicenter phase II clinical study in 2002^20^ and was approved for use in GIST by the EMA and FDA successively. Imatinib, which has alternate names, Glivec/Gleevec, was under development by Novartis Pharma AG in Switzerland.^21^ It received FDA approval for expanded use in patients with rare GIST until January 31, 2012 and was systematically coded as chemotherapy in the SEER database in 2012 [SEER*Rx Interactive Antineoplastic Drugs Database (cancer.gov)]. Few studies have reassessed the effect of imatinib on survival in the population over time. Currently, as TKI use has become widespread and TKIs are normalized into public databases, it is significant to construct a new GIST prognostic model. Based on these data, a relatively comprehensive analytical pattern can be established and graphically visualized using a nomogram. Nomograms produce measurable predictions by adding the scores of the predictors and are expected to help specific patient identification and stratification.

We derived and internally validated the nomogram from GISTs registered between 2013 and 2016 in the SEER database (updated: November 26, 2022), which were collected after the approval and coding of imatinib. Furthermore, we tested it in an external patient cohort. We retrospectively assessed pathologic elements and incorporated clinical dispositions simultaneously. The analysis procedure extracted the important endpoint, namely OS. Patients during this period were followed up for more than 5 years, and therefore, we studied the survival of patients at 1, 3, and 5 years. The presentation of the pattern was assessed and verified internally and externally from different perspectives. The survival of the patient population was reevaluated during this period when imatinib was used, imatinib use was recorded, and the patients administered imatinib were followed up regularly. This predicting model is among the latest investigations aimed at identifying patients who are more likely to potentially show poor outcomes with established practices. We hope our findings will improve the prognosis prediction of GIST and inform the selection of a treatment strategy.

## 2 MATERIALS AND METHODS

### 2.1 Patient selection and variable introduction

Data of patients enrolled into the database during 2013–2016 were captured using the SEER*Stat software (version 8.4.0.1, https://seer.cancer.gov/seerstat/, updated on November 26, 2022). We acquired written approval and consent to obtain and use the information on clinicopathological features and survival. We adhered to the arrangement throughout the study (accession number: 19787‐Nov2021). To identify the eligible participants, the following inclusion criteria were applied: (1) tumor site: digestive system (ICD‐O‐3/WHO2008), (2) histology: 8935–8936 (ICD‐O‐3), (3) initial diagnosis year: 2013–2016, and (4) available data on OS. The exclusion criteria were as follows: (1) diseases confirmed only from a death certificate or on autopsy, (2) the presence of other primary cancers besides GIST, (3) patient age <18 years, and (4) missing critical information (e.g., surgery and positive histology).

**FIGURE 1:**
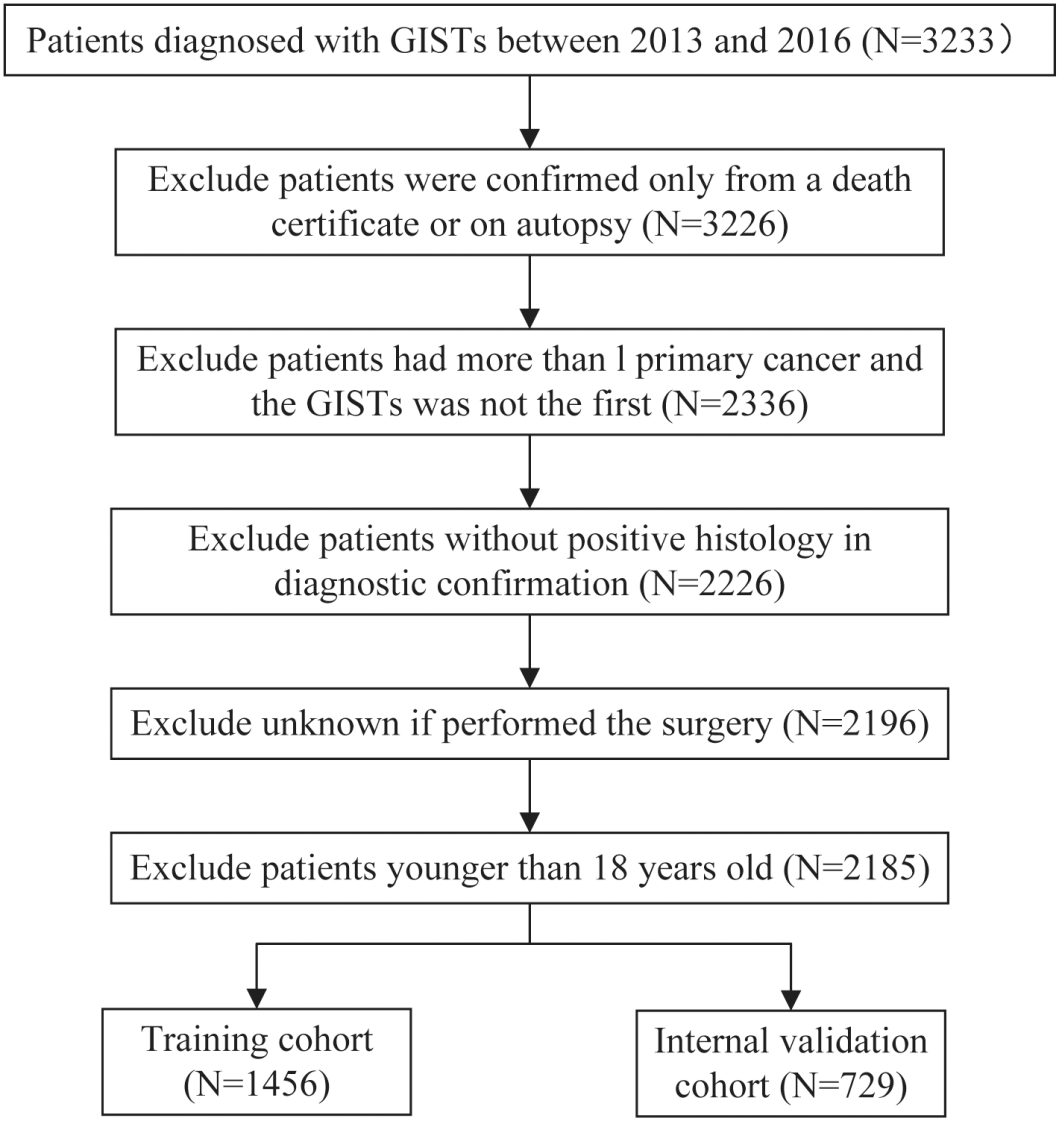
Flow diagram of the data selection process from SEER database constructing training and validation sets.

Information on clinicopathological features and survival for 3233 cases was obtained from the SEER database. We excluded cases where GISTs were confirmed from a death certificate or during autopsy,^7^ cases where GIST was not the only or the primary illness (890), and cases with missing essential information (140) or those not meeting the age criteria.^11^ After these exclusions, the study finally comprised 2185 patients with GIST. These patients were randomly assigned to a training cohort (*n* = 1456) and a validation cohort (*n* = 729) in a 2:1 ratio. The data filtering process is shown in Figure 1. In addition, to test the generalizability of the model, we reviewed 159 patients who comprised our external validation cohort; these patients received a pathological diagnosis of GIST between January 1, 2015 and June 30, 2017 in Xijing Hospital. The inclusion criteria for these patients were the same as those for inclusion in the database. As all data in the SEER database were publicly available, the need for ethical approval was waived. Externally validated subjects provided written informed consent. Data on important items were gathered, such as age, gender, marital status, tumor size, the mitotic index, primary tumor location, summary stage, cancer‐directed surgery, chemotherapy, the TNM7th stage, and follow‐up outcomes. OS, which was the endpoint, was defined as the interval between identification and death or last follow‐up, regardless of the cause.

### 2.2 Statistical analysis

On the basis of the NCCN guidelines (Version 2.2022, Gastrointestinal Stromal Tumors [GISTs]), we modified continuous variables, such as age, the mitotic index, and tumor size, into categorical data referring to admitted thresholds. After excluding patients aged <18 years, the cutoff value for age was 60 years, which was used to bifurcate and group people with age at diagnosis of ≤60 and >60 years. If the required information was absent, categorical variables were used as dummy variables. The mitotic index was parted as ≤5 and >5mitoses per 50 HPF (40× fields) and unknown. The tumor size groupings were ≤5, 5–10, >10 cm, and unknown, respectively. Categorical data were exhibited as frequencies and proportions in three cohorts and were analyzed using the chi‐square test. Items identified as statistically significant in the univariate Cox regression analysis were subjected to multivariate analysis using training data. Multivariate Cox regression analysis was used to individually evaluate the association of all elements with OS by calculating hazard ratios and 95% CIs. Significant items (*p* < 0.05) were considered as the independent predictors. The nomograms were drawn with these independent items. Probabilities of 1‐, 3‐ and 5‐year OS were derived by collating the scores of all the indicators.^22^ To further facilitate the use of the model, the model was visualized into a graphical web page wherein OS could be predicted by entering relevant elements.^23^

The model was evaluated (training cohort) and verified internally (validation cohort). We used the patient information collected at clinical follow‐ups for further external validation (external cohort). The concordance index (C‐index) was calculated to evaluate the discriminatory power.^24^ A higher C‐index suggested a higher degree of discrimination. The receiver operating characteristic (ROC) curves, which indicate the sensitivity and specificity of the model, were operated to further estimate the discrimination. A larger area under the ROC curve (AUC) was associated with better judgment ability.^25^ The calibration curves illustrated the calibration between factual and model‐estimated outcomes. A plot along the 45‐degree line denoted excellent accordance, with the projected probability matching the factual outcome.^26^ One thousand bootstrap resamples were available for correcting the C‐index for a relatively unbiased estimate.^27^ Furthermore, we compared the model with the TNM7th staging system by analyzing the predictive power. Statistical analyses were performed using SPSS version 23.0 and R version 4.1.0 software in R Studio. A *p* value descriptive data were generated for every key node in the analysis procedure. A *p* value of <0.05 was considered significant.

## 3 RESULTS

### 3.1 Demographic and clinical features

Herein, 2185 patients diagnosed with GISTs between 2013 and 2016 were enrolled in the study from the SEER database. Table 1 shows the principal components and intercorrelations among the three cohorts. These patients were randomly divided in a 2:1 ratio—1456 patients in a training cohort and 729 in the validation cohort. The median age of patients was 63 years (range, 19–98 years). The proportion of married individuals overall was 56.8%. Approximately 63.0% of the lesions were located in the stomach. In terms of tumor dimensions, most tumors were <5 cm (40.6%), followed by those measuring 5–10 cm (27.7%) and >10 cm (23.4%). The diameter was unknown for 8.3% of tumors. In 47.0% of tumors, the mitotic rate was >5 mitoses/50 high‐power fields. The summary stage, which indicates how far the tumor has spread from its point of origin, for the majority was localized (1439; 65.9%), followed by distant (407; 18.6%), and regionalized (234; 10.7%). The proportion of patients having cancer‐directed surgery was 81.7%. Notably, 48.1% of the patients received chemotherapy. The median OS was 49 months (range, 0–83 months) in the training cohort and 51 months (0–83 months) in the validation cohort. Notably, 338 (23.2%) patients in the training cohort died during the follow‐up period. The correlation between the training and the internal group was checked using the chi‐square test, and the results were not significant (*p* > 0.05).

**TABLE 1:** Patient demographics and pathological information.

Variables	All patients (*n* = 2185)		Training cohort (*n* = 1456)		Internal cohort (*n* = 729)		External cohort (*n* = 159)	
	Numbers	%	Numbers	%	Numbers	%	Numbers	%
Age (years)								
≤60	965	44.2	641	44.0	324	44.4	78	49.1
>60	1220	55.8	815	56.0	405	55.6	81	50.9
Marital status								
Married	1241	56.8	819	56.3	422	57.9	80	50.3
Unmarried^a^	836	38.3	568	39.0	268	36.8	59	37.1
Unknown	108	4.9	69	4.7	39	5.3	20	12.6
Tumor size (cm)								
≤5	888	40.6	588	40.4	300	41.2	66	41.5
5–10	605	27.7	386	26.5	219	30.0	41	25.8
>10	511	23.4	347	23.8	164	22.5	27	17.0
Unknown	181	8.3	135	9.3	46	6.3	25	15.7
Mitotic index (mitoses per 50 HPF)								
≤5	592	27.1	386	26.5	206	28.3	85	53.5
>5	1028	47.0	680	46.7	348	47.7	45	28.3
Unknown	565	25.9	390	26.8	175	24.0	29	18.2
Surgery								
Yes	1786	81.7	1180	81.0	606	83.1	117	73.6
No	399	18.3	276	19.0	123	16.9	42	26.4
Chemotherapy								
Yes	1052	48.1	719	49.4	333	45.7	79	49.7
No/Unknown	1133	51.9	737	50.6	396	54.3	80	50.3
Summary stage								
Localized	1439	65.9	942	64.7	497	68.2	66	41.5
Regional	234	10.7	157	10.8	77	10.6	41	25.8
Distant	407	18.6	282	19.4	125	17.1	30	18.9
Unknown/Unstaged	105	4.8	75	5.2	30	4.1	22	13.8
Primary site								
Stomach	1376	63.0	900	61.8	476	65.3	106	66.7
Nongastric^b^	809	37.0	556	38.2	253	34.7	53	33.3

^a^ Single, Divorced and Separated, or Widowed.

^b^ Other parts of the digestive tract.

The median age at diagnosis of 159 patients from Xijing Hospital who were incorporated as the external cohort was 59 years (25–84 years). Most were married (50.3%), and a proportion of them refused to disclose their marital status (12.6%). In terms of tumor dimensions, most were <5 cm (41.5%), followed by those measuring 5–10 cm (25.8%) and > 10 cm (17.0%). The mitotic index of most tumors (53.5%) was <5 mitoses/50 high‐power fields, and among the stages, localized tumors were noted in a marked proportion of the cases (41.5%). Primary GISTs were the most common in the stomach (66.7%), which is consistent with previous reports. Others were localized to other parts of the digestive tract. Notably, 73.6% of patients underwent surgery on the primary site and 49.7% received chemotherapy.

### 3.2 Predictive nomogram

**FIGURE 2:**
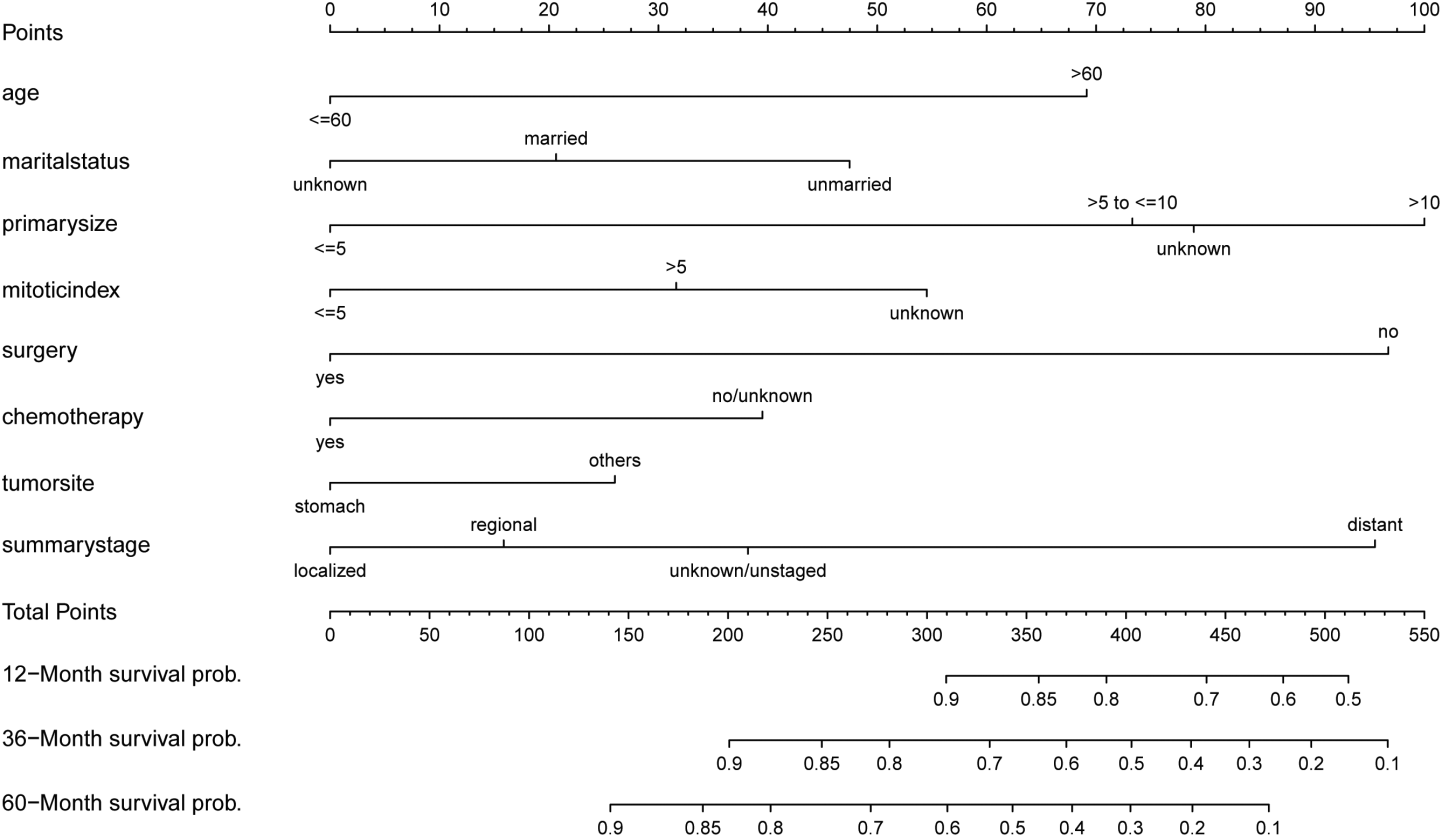
Nomogram for predicting 1‐, 3‐, and 5‐year overall survival of patients with GIST. Marital status: unmarried means single, Divorced and Separated, or Widowed; Primary site: nongastric means other parts of the digestive tract. Units of measurement: age (years old), tumor size (cm), mitotic index (mitoses per 50 HPF).

**FIGURE 3:**
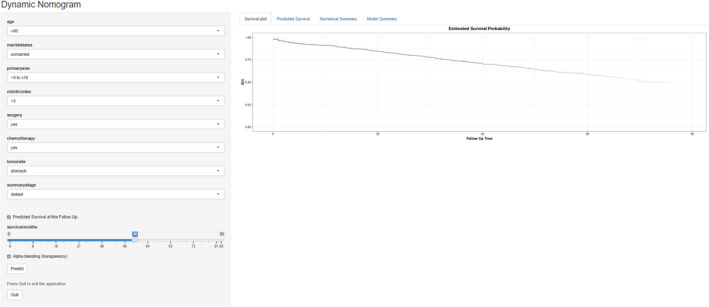
A screenshot of the model as a web version. Marital status: unmarried means single, divorced and separated, or widowed; primary site: nongastric means other parts of the digestive tract. Units of measurement: age (years old), tumor size (cm), mitotic index (mitoses per 50 HPF).

Univariate analysis revealed that 8 parameters (age, marital status, tumor size, the mitotic index, cancer‐direct surgery, chemotherapy, summary stage, and primary site) were significant predictors of OS. As shown in Table 2, multivariate analysis revealed the following independent prognostic indicators: age, tumor size, the mitotic index, surgery, chemotherapy, summary stage, and primary site. These independent predictors were integrated to establish a nomogram predicting 1‐, 3‐, and 5‐year OS (Figure 2). The dynamic nomogram for OS is shown in the webpage form (Dynamic Nomogram [shinyapps.io]). After entering variable information on this web page, the dynamic histogram showed the calculated survival probability and generated associated numbers, tables, and corresponding survival plots (Figure 3).

**TABLE 2:** Univariate and multivariate analyses of overall survival (training cohort).

Variables	Univariate analysis	Multivariate analysis	
	*p* Value	HR (95% CI)	*p* Value
Age (years)	<0.001*		
≤60		Reference	
>60		1.898 (1.508–2.389)	<0.001*
Marital status	0.024*		
Married		Reference	
Unmarried^a^		1.282 (1.029–1.597)	0.027*
Unknown		0.825 (0.474–1.437)	0.497
Tumor size (cm)	<0.001*		
≤5		Reference	
5–10		1.975 (1.408–2.772)	<0.001*
>10		2.528 (1.787–3.575)	<0.001*
Unknown		2.075 (1.376–3.130)	0.001*
Mitotic index (mitoses per 50 HPF)	<0.001*		
≤5		Reference	
>5		1.342 (0.954–1.887)	0.091
Unknown		1.658 (1.130–2.433)	0.010*
Surgery	<0.001*		
Yes		Reference	
No		2.452 (1.801–3.338)	<0.001*
Chemotherapy	<0.001*		
Yes		Reference	
No/Unknown		1.436 (1.118–1.845)	0.005*
Summary stage	<0.001*		
Localized		Reference	
Regional		1.159 (0.768–1.748)	0.482
Distant		2.423 (1.769–3.319)	<0.001*
Unknown/Unstaged		1.430 (0.881–2.321)	0.148
Primary site	<0.001*		
Stomach		Reference	
Non‐gastric^b^		1.274 (1.017–1.595)	0.035*

^a^ Single, Divorced and Separated, or Widowed.

^b^ Other parts of the digestive tract.

* Statistically significant.

### 3.3 Nomogram validation

**FIGURE 4:**
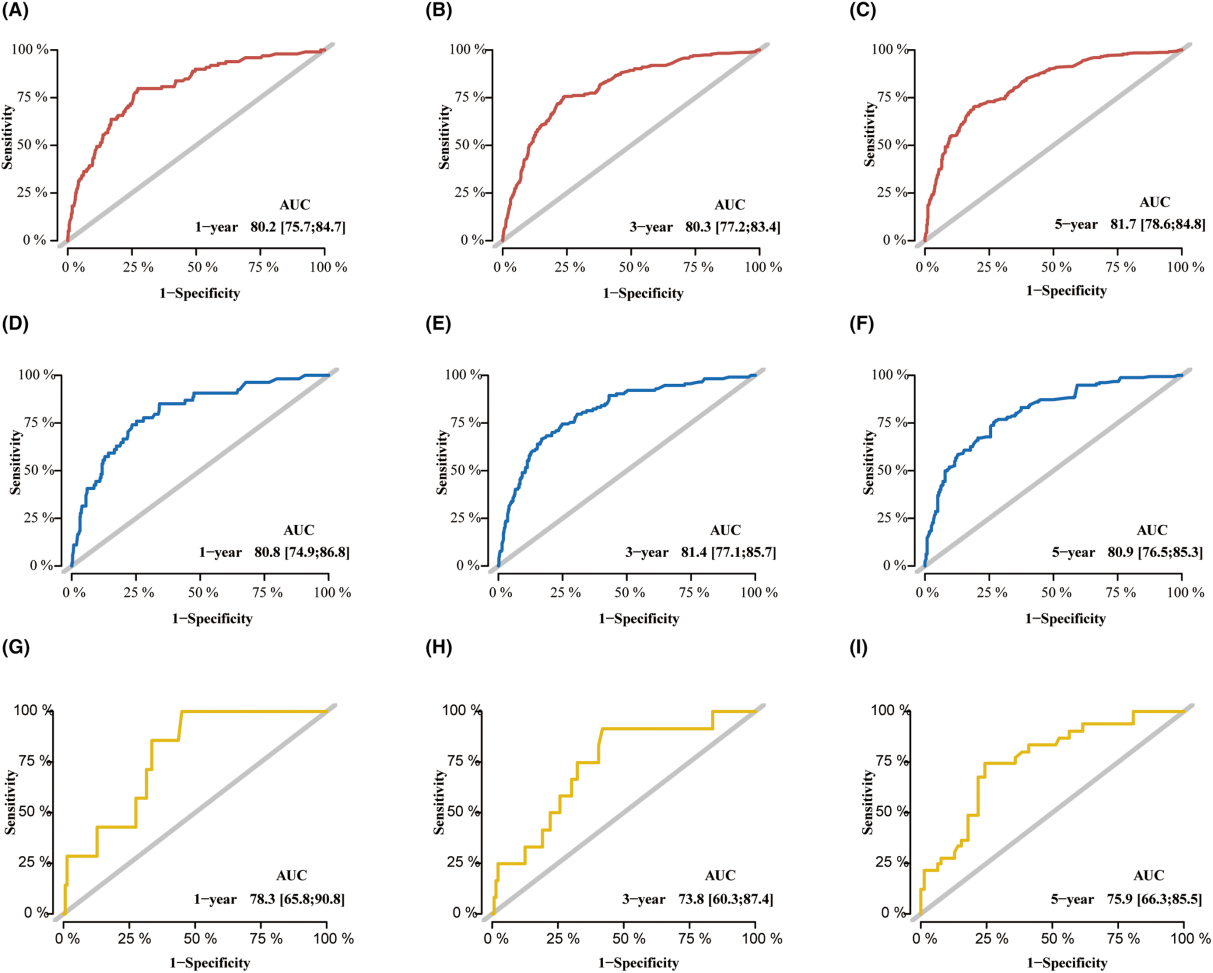
The receiver operating characteristic curves of the nomograms for 1‐, 3‐, and 5‐year overall survival in the training cohort (A–C), validation cohort (D–F), and external cohort (G–I).

**FIGURE 5:**
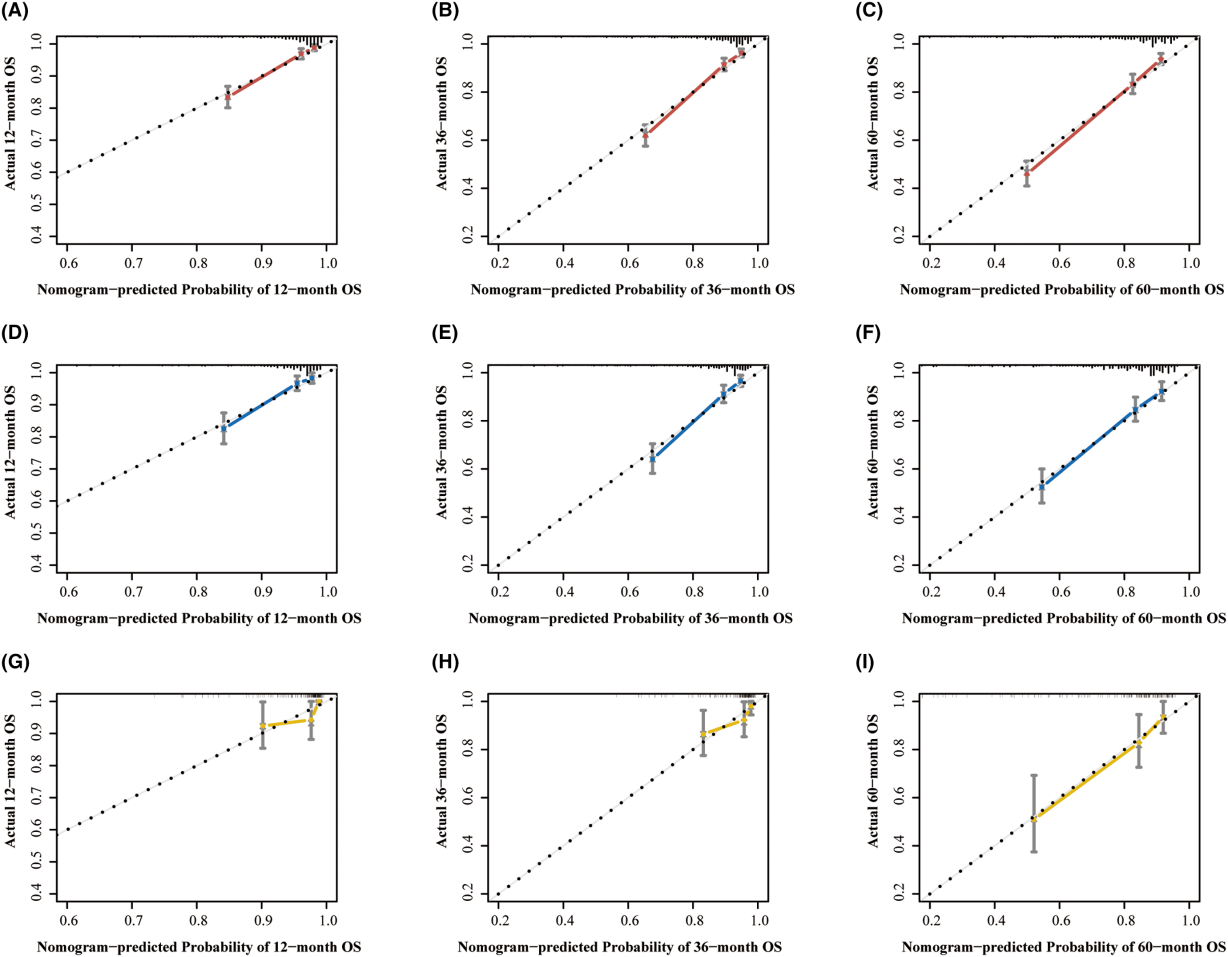
Calibration plots of the nomogram for 1‐, 3‐, and 5‐year overall survival (OS) in the training cohort (A– C), validation cohort (D–F), and external cohort (G–I). The vertical bars represent 95% confidence intervals.

**FIGURE 6:**
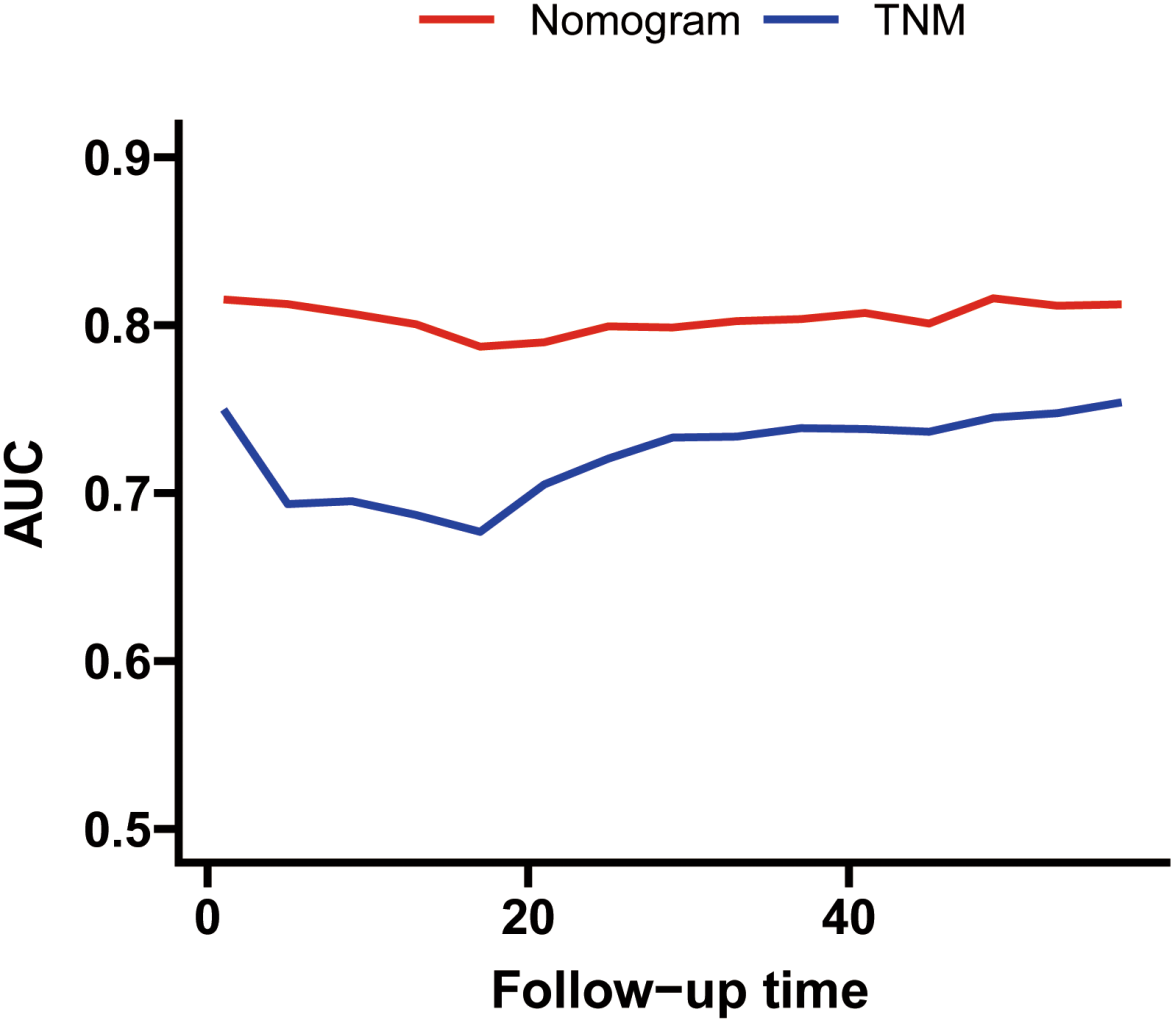
Area under the ROC curves for the nomogram models versus TNM models along the follow‐up.

The nomograms were evaluated and observed to have good performance in internal and external validation cohorts. The concordance indices are shown in Table 3. The C‐index of the model was 0.777 (95% CI, 0.752–0.802), indicating good concordance. The original C‐index in the internal validation cohort was 0.7787. After applying the bootstrap method, it was corrected to 0.7785. The model was generalized to the cohort of locally collected patients for further external testing. The original C‐index in the external cohort was 0.7613 and corrected to 0.7579 after applying the bootstrap method. ROC showed the AUCs for OS in 1, 3, and 5 years were 0.802, 0.803, and 0.817 in the training cohort, and 0.808, 0.814, and 0.809 in the internal validation cohort. The 1‐, 3‐, and 5‐year AUCs in the external validation cohort were 0.783, 0.738, and 0.759, respectively (Figure 4). The calibration plots of OS in three cohorts indicated satisfactory correspondence between predictions and the exactly noted outcomes (Figure 5). Together, these results provided important insights into the nomograms' good prediction capability. Upon comparing the nomograms and the TNM models, it could be seen that the discrimination of the model was better than that of TNM7th stage for OS prediction. The C‐index was computed as 0.777 (95% CI, 0.752–0.802) for the nomogram versus 0.716 (95% CI, 0.689–0.743) for TNM7th staging (Table 3), thus upholding the superiority of this model. Interestingly, the superiority of the predictive model over the TNM staging was further upheld by AUC plots (Figure 6). The AUCs for OS were significantly better for the nomograms than the TNM7th staging along the follow‐up. Because lymph node involvement in GISTs is rare and factors included in the TNM staging are inadequate, risk stratification was often used instead of TNM staging in clinical practice, and these data were missing in the external cohorts.

**TABLE 3:** C‐indices for the models and the TNM7th staging system.

Survival		Training cohort		Validation cohort		External cohort	
		C‐index	95% CI	C‐index	C‐index corrected	C‐index	C‐index corrected
Overall survival	Nomogram	0.777	0.752–0.802	0.7787	0.7785	0.7613	0.7579
	TNM 7th staging system	0.716	0.689–0.743	0.7142	0.7148		

Abbreviations: C‐index, concordance index; CI, confidence Interval.

## 4 DISCUSSION

The emergence of imatinib as a representative TKI laid the cornerstone of GIST precision treatment and affected the prognosis of patients with GIST globally. Although most patients (about 60%) with operable GIST can be completely cured by surgery,^28^ advanced cases are unsuitable for surgery at the outset. For larger tumors, they are prone to rupture or carry a risk of damage to vital adjacent organs during surgery. In some patients with severe metastases and relapse, when disease progression is identified, expanding the scope of surgery does not improve the cure rate. These individuals are particularly suitable for receiving neoadjuvant chemotherapy and can be given a combination of neoadjuvant chemotherapy and surgery as the complete radical tumor treatment. Imatinib is the first‐line treatment, which was reported to dramatically prolong the survival of patients with GIST. In a randomized, open‐label, multicenter phase II clinical trial for imatinib, 147 advanced GISTs randomly classified in a 1:1 ratio were treated with daily oral imatinib 400 mg or 600 mg.^20^ The results showed that 79 patients achieved partial response (53.7%) and 41 patients had stable disease (28%). Overall response rates (ORR), median progression‐free survival (PFS), and OS were comparable between the two doses. The median OS for all patients was 57 months.^29^ Conversely, before imatinib, patients with metastatic GISTs had a median OS of roughly 20 months, and in cases of local recurrence, patients had a median OS of only 9–12 months.^30^ In neoadjuvant treatment, patients with giant localized tumors should be given neoadjuvant TKI therapy before surgery because tumors can be too complicated to be removed due to adjacent vital organ involvement or excessive surgical risk. Two‐year PFS was 88.4% in the surgery for the residual disease arm and 57.7% in the Imatinib‐alone arm (*p* = 0.089).^31^ On CT imaging, treatment benefit can be shown as a reduced tumor density, and the maximal reduction in size may take up to 6 months.^32^ When the condition is stable with TKIs or disease progression is significantly inhibited, radical or debulking surgical resection may be considered. In response to impressive results, after imatinib was widely used and normalized into public databases as chemotherapy, several expositions in statistical prediction become unsatisfactory. The evaluation system needs updating.

More critically, having the correct judgment of a patient's condition can not only help predict the prognosis but also play an important role in the treatment process. Imatinib is remarkable, but owing to the KIT gene secondary mutation, about half of cases will develop resistance to imatinib after 2 years on the drug.^33^, ^34^ Although dose adjustments and subsequent generations of TKIs like sunitinib and regorafenib are still used after developing imatinib resistance, the benefit is limited. About 2% of patients respond to a dose increase from 400 mg/day to 600 mg or 800 mg/day. The PFS after imatinib dose escalation was 5.1 months.^35^ The ORRs of sunitinib^36^ and regorafenib^37^ are approximately 5%–7%, and the median PFS is no more than 6 months. The latest avapritinib and ripretinib are effective and selective TKIs, which have been approved both internationally and in China for the treatment of PDGFRA 18 exon mutation and fourth‐line metastatic gastrointestinal stromal tumors, respectively. Recently, phase III clinical studies on the simultaneous use of avapritinib and ripretinib have been carried out. In the third‐line treatment of metastatic GIST with avapritinib, it showed a higher ORR than regorafenib but did not further improve PFS, specifically in patients with the D842V mutation.^38^ In a phase III study comparing ripretinib and sunitinib for the second‐line treatment of metastatic GIST, although ripretinib showed a dominant trend in GIST with primary exon 11 mutation, it failed to further improve PFS in the overall population.^39^ In a nutshell, two new drugs failed to change the overall pattern of metastatic gastrointestinal stromal tumor treatment. Due to medication particularity and insidious symptoms, close monitoring is essential during their administration. Patients who no longer benefit from medication alone need to be selected for prompt surgical intervention. GISTs in some patients can rapidly become unresectable and lead to disease progression. In light of such conditions, updating and optimizing the evaluation of subsequent outcomes has garnered increased attention. Identification of whether a patient will get longer OS from the surgical resection or continuous imatinib treatment is an urgent requirement.

Previous studies were based on data before the admission with limited factors included and have thus failed to address how imatinib helps prolong the OS. This paper included clinicopathological features and dispositions since imatinib was registered in the database. We built models aimed to predict OS at different points of time, validated the models, and visualized their performance. This study provides an updated understanding of the link among pathological features, dispositions, and survival. The predicting model establishment was derived from independent predictors. In multivariate Cox regression analyses, age clearly emerged as an independent prognostic factor for the primary endpoints with older individuals showing relatively poor prognosis, which is in line with several previous researches.^40^ Studies have reported an association between marital status with OS as well.^41^, ^42^ Married individuals were likely to get a better prognosis as per our findings. This could be explained by married people being more likely to be guarded and have better medication adherence.^43^, ^44^ In addition, tumor size, the mitotic index, and location were all identified as principal determiners, which is in line with the wide recognition of their significance.^45^ Cutoff values for tumor size and the mitotic index were decided according to the NCCN guidelines. For both these indicators, incomplete information was associated with a relatively poor prognosis predicted by the model, which also suggests the significance of standardized and complete examination. Tumors were identified as gastric and nongastric tumors as per the NIH criterion, and nongastric tumors were associated with a higher risk, as reported.^46^ The model also incorporated important implications associated with tumors from diverse primary sites into prognosis prediction. The significance of the primary site of the tumor was reported by Blay et al.^47^ Summary stage was another important predictor. Due to the mesenchymal origin of GIST, localized, regional, and distant tumors are included in the model instead of tissue infiltration like in other cancers, which also contributed to our model's superiority to the TNM staging system. Of particular importance is that cancer‐directed surgery and TKI therapy weighed heavily in the models. The graph suggested that patients with surgery have significantly longer PFS than those without surgery(*p* = 0.089), which corroborates Du et al.'s report.^31^ In a pooled analysis of data from 10 population‐based series comprising 2459 patients, only a few postoperative tumors recurred after the first 10 years of follow‐up, indicating that surgery is likely to treat most patients with operable GIST (about 60%).^28^ Imatinib is the preferred TKI for the first‐line medication treatment, as per the database. Using a robust data source, the significance of imatinib for the prolonged survival of patients with GIST was fully demonstrated here. Although we could not include all of the possible criteria, we ensured that the items that finally made up the model were significant. From the C‐index reckoned internally and externally and from ROC curves and calibration plots, the model indicated good discrimination and calibration abilities. The conclusion was consolidated while testing. Moreover, the model performed showed better discriminatory power than the TNM7th staging system thanks to supplementary parameters. This higher performance is reflected in the AUCs of the models. The prediction model was verified in the external cohort, and the C‐index and AUC further prove the satisfactory accuracy and discrimination power of the model.

Nomograms are statistical tools that can indicate the possibility of survival for a given outcome through a simple diagram. Some previous evidence suggests that in many malignancies, their use has gradually been recognized as an alternative to the TNM staging system or even considered a new standard.^48^ We systematically gathered the demographic and clinicopathological information of patients with GIST. Reliable and rational processing of data guaranteed the accurate establishment of the predictive model. The nomograms were well verified and expressed better predictive capability than the TNM7th staging system. The data required for creating a nomogram are easily available in clinical practice. For application convenience, a webpage‐based dynamic nomogram was developed to dynamically predict the prognosis. It is simple to use and does not require any registration permissions or passwords.

Despite the study being based on a large population, some limitations were unavoidable. First, retrospective data constraints may lead to a potential for selection bias. Although external validation was performed, the external cohort had a small sample size and fewer patients with outcome events after 5 years of follow‐up and some potential for data deviation. It may also be the cause of excessive standard deviation in the calibration curve, particularly for 5‐year survival. In the future, it is necessary to continue to collect cases, increase the sample size, and improve the model. Second, this study could not include all items likely to contribute to the OS, such as preoperative or intraoperative tumor rupture, which is associated with a significantly increased risk of recurrence. Occult peritoneal illness can exist due to the spilling of tumor cells into the peritoneal cavity when a tumor ruptures. The patients are subject to high risks upon peritoneal relapse.^49^ Thus, it is regrettable that this information was not provided in the database. In addition, GIST is a highly heterogeneous group of tumors with multiple types of oncogene mutations activated. All subtypes have different molecular driver changes and different natural histories. Specific molecular alterations in various oncogenes associated with tumor progression make a significant difference in terms of efficacy and response to different drug treatments, particularly imatinib. Primary resistance to imatinib exists in GISTs with PDGFRA D816V and D842v mutations, which account for almost half cases with PDGFRA alterations.^50^ In advanced cases, the PFS achieved with imatinib is shorter for GISTs with KIT exon 9 mutations (12.6–16.7 months) than for GISTs with KIT exon 11 mutations (usually now >24 months). The median OS was 55 months for patients with KIT exon 9 mutations. As for KIT exon 11, the median OS was more than 73 months in the median follow‐up.^51^ Tumors with KIT exon 11 mutations at codon 557/558 are more aggressive and carry a higher risk of recurrence and metastasis.^52^ While receiving sunitinib, the second‐line treatment, GISTs with exon 9 mutation are associated with a longer PFS than those with exon 11 mutation, with median PFS of 12.3 and 7.0 months, respectively.^53^ The presence and type of KIT or PDGFR mutations should be identified and evaluated as a predictor of response to imatinib. Genetic mutation status should be identified before therapy selection. However, genotyping data have not yet been enrolled into the database, which may be due to a relatively low prevalence.^54^ Third, the importance of tumor size in predicting prognosis is closely related to primary sites. Rectum GIST tends to have aggressive biological behavior, and tumors with mitotic activity can recur and spread despite a small size of <2 cm.^55^ When GISTs from different sites are considered together, the accuracy of the model may be affected. Fourth, details about the clinical course are lacking. Some people undertaking TKIs have certain adverse reactions^56^ and affect patients' medication compliance to some extent.^57^ The time point at which patients with prolonged medication developed resistance is also important. Once the withdrawal happens, these statuses may become significant risk factors related to survival. With the lack of information on these abovementioned aspects, the model still needs further improvement, and this will be the focus of our intensive research in the future. It is more sensible to treat the nomogram as a reference providing a potential outcome while evaluating clinical options.

## 5 CONCLUSION

We aimed to develop a comprehensive survival prediction model for assessing the 1‐, 3‐, and 5‐year OS of patients with GISTs in the postimatinib era. The new nomogram expressed satisfactory presentation in training, internal validation, and external validation cohorts and outperformed the traditional TNM staging systems. The visualized model can be considered an authentic tool for providing a potential outcome. Nevertheless, there is still a definite need for more clinical information to incorporate more risk factors and improve the model. Randomized clinical trials remain the gold criterion in testing.

## AUTHOR CONTRIBUTIONS

**Shu Wang:** Data curation (lead); resources (lead); writing – original draft (lead). **Yuhao Wang:** Formal analysis (equal); methodology (equal). **Jialin Luo:** Data curation (supporting); formal analysis (equal). **Haoyuan Wang:** Validation (equal). **Yan Zhao:** Visualization (equal). **Yongzhan Nie:** Conceptualization (equal). **JianJun Yang:** Conceptualization (equal); funding acquisition (lead).

## FUNDING INFORMATION

This work was supported by the National Key Research and Development Program of China (2020YFE0202200), the National Natural Science Foundation of China (82172973), and the Key Research and Development Project of Shaanxi Province (2022ZDLSF03‐04).

## CONFLICT OF INTEREST STATEMENT

The authors declare that they have no competing interests.

## ETHICS APPROVAL AND CONSENT TO PARTICIPATE

Data from the SEER database were obtained in a public manner, no ethical approval is required. Subjects for externally validated have signed informed consent. Ethics approval has been obtained from the Ethics Committee at the First Affiliated Hospital (Xijing Hospital) of Fourth Military Medical University (KY20213386‐1). Informed consent is obtained from all study participants.

## CONSENT FOR PUBLICATION

Not applicable.

## Data Availability

The data in this research were retrieved from an open public database and can be accessed through this link: https://seer.cancer.gov/.
